# Linguistic–Cultural Mediation in Asylum and Refugee Settings and Its Emotional Impact on Arabic–Spanish Interpreters

**DOI:** 10.3390/ejihpe11040093

**Published:** 2021-10-18

**Authors:** Bachir Mahyub-Rayaa, Moulay-Lahssan Baya-Essayahi

**Affiliations:** Department of Translation and Interpreting, University of Granada, 18002 Granada, Spain; baya@ugr.es

**Keywords:** linguistic–cultural mediation, emotional impact, negative emotions, Arabic–Spanish interpreting, asylum, refuge

## Abstract

Emotional factors in linguistic–cultural mediation have attracted the attention of prior literature for a number of decades, both regarding the cognitive processes involved in language interpreting and the impact of stress and emotion on the performance of interpreters. However, research has not yet been replicated in the Arabic–Spanish pair, despite it being one of the combinations most requested by public services in Spain and Europe. Methodology: An exploratory study conducted by an anonymous online 17-item survey was carried out in order to discover the perception of Arabic–Spanish interpreters in asylum and refugee settings about the emotional impact of their job. Out of 30 contacted, 23 active interpreters completed the survey. Results: The answers showed that all of the interpreters had been exposed to situations that had emotionally impacted them. Triggering situations and a list of negative emotions were collected. Discussion: Direct and indirect implications of the referred emotional episodes and their consequences on the performance of the interpreters were analysed and discussed. Conclusions: Linguistic–cultural mediation in these settings exposes interpreters to harsh stories that trigger mostly negative emotions. These professionals lack psychological support; thus, they are forced to deal individually with each situation, without taking into account the possible consequences on their work and their physical and mental health.

## 1. Introduction

The existence of emotions has been accepted by psychology from the times of Darwin [1,2] up to the more contemporary eras of Tomkins [3,4], Ekman and Friesen [5] and Izard [6]. Thus, the importance of their adaptive function for the survival of human beings and the species in general has been highlighted from the beginning. As the protagonist of the novel *The Portrait of Dorian Gray* once said: “I don’t want to be at the mercy of my emotions. I want to use them, to enjoy them, and to dominate them”.

Within the framework of the study of language interpreting or language and cultural mediation (terms that shall be employed synonymously in this work given the essence of this profession in asylum and refugee contexts), the impact of emotions on the cognitive process required by this interlinguistic and intercultural task has attracted the attention of research for a number of decades. The focus of interest was initially on neurology in order to gain an understanding of the cognitive and physiological mechanisms that intervene when the words of a speaker are simultaneously interpreted from one language to another in milliseconds over an extended period of time. Authors such as Gerver [7], Seleskovitch [8] and Chernov [9], among others, were therefore interested in the underlying cognitive process in the transfer between two languages and in the duality of tasks (listening and speaking) that the exercise of interpreting requires. Later, there was the emergence of Gile’s “Effort Model” [10], which feeds the cognitive theory of interpreting and explains how the cognitive efforts that intervene in conference interpreting are managed [11]. More recently, there have been studies that analyse the effect of stress and emotions in the exercise of this interpreting [12,13]. An interesting contribution as regards the psychological and emotional impact on public service interpreters and translators can be found in Márquez Olalla [14] and in the review offered by Valero-Garcés [15], despite over a decade having passed since. In line with the aim of this work, the study carried out by Baistow [16], “the Emotional and Psychological Impact of Community Interpreting”, is especially interesting, as well as more general works dealing with different professional aspects of Arabic/Spanish interpreting [17].

It can be gathered from the bibliographical review that research into the emotional impact of language interpreting, mainly in combinations that include English, is considerably more advanced than research activity relating to Arabic–Spanish [18,19,20,21,22,23], among others. Although this language pairing is found to be among those that have been requested the most by public services in Spain and Europe since the end of the 20th century [24,25], taking into account the migratory flows and influx of migrants and refugees across the southern border of the old continent, research on the emotional impact of Arabic/Spanish interpreting has not accompanied this demand. This reality, together with the direct observation of the work of Arabic/Spanish interpreters in asylum and refugee contexts, has encouraged us to undertake this exploratory qualitative study in order to shed light on the possible emotional impact on interpreters in this context.

### The Theory of Emotions and the Possible Psychological Impact of the Interpreted Communicative Act

Previous literature defines emotions as psychophysical responses originating in the limbic system experienced by people in the face of specific situations, memories, or experiences involving danger, harm, surprise, etc. The limbic system, in this regard, creates three types of reactions against the impact of such stimuli: physiological (physical and involuntary), cognitive (conscious and unconscious) and behavioural (changes in conduct). For Piqueras Rodríguez et al. [26] (pp. 86–87), these reactions, which are universal and, to a great extent, independent from culture, produce changes in the affective experience (cognitive–subjective dimension), physiological activation (physiological–adaptive dimension) and expressive behaviour (behavioural–expressive dimension).

When it comes to their classification, the theory of emotions distinguishes between seven types of emotions or feelings: primary, secondary, positive, negative, ambiguous, static, social and instrumental [27]. Primary or basic emotions are those experienced as a result of a direct stimulus. Secondary emotions are those acquired through social interaction [6]. Within the basic group, we can distinguish between positive and negative emotions [3,4]. Ekman [27] concluded that there are six basic important universal emotions: anger, disgust, sadness, fear, joy and surprise. Fear or anxiety, anger or rage, sadness or depression and disgust are emotional reactions based on negative affective experiences and are characterised by a high physiological activation [26] (p. 86). In this context, emphasis has also been placed on the existence of a negative affectivity, as a feature that reflects the tendency to experience negative emotions over time [28,29]. In short, the area of psychology considers that basic emotions exist in all individuals independently of their cultural origin. Their substratum is on the whole biological and they manifest themselves in communication with others and, in turn, can act as powerful behaviour motivators [30,31].

If we take into consideration that Arabic–Spanish interpreters in asylum and refugee contexts work to facilitate communication by expressing extremely harsh accounts from one language to another—as shall be seen below in the results of this study—it is to be expected that they are affected to a greater or lesser degree and, as a result, their cognitive–subjective, physiological–adaptive and behavioural–expressive capacities are likewise affected when emotions, be they negative or positive, interfere in the interpreted communicative act [32]. As indicated, interpreting or mediation from one language to another supposes an already demanding cognitive task, comprising various efforts that are interconnected and sometimes simultaneous. To sum up, these are: analytical hearing of the original message, decoding of the message/mental translation, memory, taking and subsequent reading of notes (when used) and reformulating in the target language, all under a primordial effort of coordination that is essential to avoid undermining the process (see Gile’s “Effort Model” theory [10]). Given this demanding task, which can reach the limit of mental fatigue, it is to be expected that when other external emotional stimuli are added to it (i.e., harsh account of an asylum seeker), the interpretive exercise is compromised [15]. In this regard, it should be remembered that one such stress factor is behavioural and cognitive efforts made to face these stimuli [33,34].

Lastly, it is worth highlighting that emotions can manifest themselves in non-verbal behaviour through the body or facial language of those who experience them, whether consciously or unconsciously [35,36]. This, taken to the terrain of interpreting in asylum and refugee contexts carried out in situ, may materialise in the non-verbal language of the interpreter, which could have a bearing on the account of the asylum seeker, or compromise the credibility of the interpreting itself, among other consequences.

This paper aims to analyse the perception of interpreters in asylum and refugee contexts (Arabic–Spanish working languages) of the potential emotional impact of their performance in these contexts and the strategies they apply to mitigate this impact, if any, in line with Moscoso [37], Vinogradov and Yalom [38], among others.

## 2. Research Questions and Objectives

This study is based on the premise that language interpreting in asylum and refugee contexts exposes interpreters to a high emotional impact given the type of accounts usually dealt with, and that this emotional impact can influence the performance of their work and/or their physical and mental health. This hypothesis has its origins in the experiences of two Arabic–Spanish interpreters who worked for a number of years in this context and who ended up changing their jobs due to the high levels of emotional stress they had experienced.

As well as studying this premise, the objective of this paper is to ascertain: 1. Whether Spanish/Arabic interpreting in asylum and refugee contexts has an emotional impact on the professionals that carry it out; 2. How this impact manifests itself, and where it exists; 3. What episodes lead to it; 4. What emotions it provokes and how long they last; and, finally, 5. What strategies are applied by the interpreter to face this potential emotional impact.

## 3. Materials and Methods

With the aim of analysing the starting premise and reaching the objectives outlined in this study, exploratory qualitative research was conducted. 

### 3.1. Participants

The 23 participating interpreters (12 men and 11 women) have an average age of 33 (σ 7.3), indicating to us that it is on the whole a young group, with high academic qualifications, the majority being specialised in Spanish/Arabic Translation and Interpreting. The studies carried out and the academic level of the group are as follows, see Table 1:

The language profiles of the participants generally lean towards Spanish as the mother tongue (A language), Arabic as the first foreign language (B language) and French or English as second foreign languages (C language). See below, Table 2:

In view of the above data, the main working language combination is Spanish/Arabic (18 interpreters), although Spanish/French is also featured (6), along with Spanish/English (2) and Spanish/Berber (1). The interpreters can work in more than one combination. The Arabic language employed should be understood as the use of the standard variety and dialects thereof.

### 3.2. Instruments

We opted to apply the anonymous survey method with Spanish/Arabic interpreters. An online survey was created with 25 initial items [39], of which only 17 are analysed here, given the limitation of the length and objective of this paper. Six open questions were posed, 5 closed, 4 open complementary to a closed question and 2 multiple choices with an open response field.

The survey is divided into three main blocks of questions (see the full version at the link indicated at the end of this paper):

- General aspects: academic and professional profile.
- Specific aspects: emotional impact of interpreting in asylum and refugee contexts.
- Specific aspects: strategies adopted to mitigate the possible emotional impact.

### 3.3. Procedure and Data Analysis

After an initial pilot using interpreters not participating in the study, and with the guidance of Álvaro Serrano Macon, expert in Psychology and Human Resources, the final version of the survey was launched on 1 October 2020 and active until 26 November of the same year. It was sent to a previously created list of 30 active interpreters working for the Spanish Commission for Aid to Refugees (Comisión Española de Ayuda al Refugiado—CEAR), the UN Refugee Agency (UNHCR) and the temporary stay centres for immigrants (CETI in Spanish), as well as freelance interpreters working for other social services either in person or by telephone. Twenty-seven completed surveys were received (90% response rate). However, 4 were rejected due to lack of experience in the context analysed, to which the valid responses finally stood at 23 (76.6% valid response rate).

In view of the scarcity of research in this context, especially regarding the Spanish/Arabic combination, the intended approach for this study is mainly qualitative, although closed yes or no questions have been contemplated where necessary. Despite the fact that the population sample is representative of the number of professionals carrying out Spanish/Arabic interpreting in this context, the motivation behind this study is not quantitative, given that we are still at an exploratory stage.

Employing an online survey has been useful and dynamic in these COVID-19 pandemic times, and in light of the restrictions imposed; nevertheless, our initial intention was to carry it out in person on an individual basis, adding a brief semi-structured interview in order to obtain more qualitative feedback.

One limitation that should be mentioned in this section is that the survey does not include questions on the emotional stability of the participating interpreters prior to or outside of their work in asylum and refugee contexts.

Once the survey stage was completed, all responses were compiled in a spreadsheet and analysed in light of the objectives set and in line with the research questions posed for this work.

## 4. Results

For the sake of clarity, we present here the main results obtained. The complete results of the survey can be consulted at Mahyub Rayaa [39].

### 4.1. Frequency of Interpreting in Asylum and Refugee Contexts

As indicated previously, all participants in the study have at some point interpreted in asylum and refugee contexts. The frequency of work in this field in general varies between daily and weekly (52.2% of interpreters). The overall results in this regard are as follows, Table 3:

### 4.2. Interpreting Techniques Used in Asylum and Refugee Contexts

The majority of interpreters indicate the use of liaison interpreting, also known as bilateral or dialogue interpreting, in face-to-face format, although a number point to the change to liaison via telephone or videoconference, especially since the declaration of the state of alarm in Spain (14 March 2020) and the lockdown imposed to combat the COVID-19 pandemic. The interpreting techniques used in asylum and refugee contexts are as follows, Table 4:

### 4.3. Emotional Impact of Interpreting in Contexts of Asylum and Refuge

To the question of whether the participants have carried out interpreting jobs in asylum and/or refugee contexts that have had an emotional impact on them, 100% responded in the affirmative.

To delve deeper into this question and obtain a possible list of trigger episodes, in the case of an affirmative response, they were asked to give a summarised and anonymous description of the cause of the impact (see [39] for whole descriptions provided by the participants). In practically all of the cases, the interpreters indicated the content of the asylum seekers’ accounts and the way in which they relate the experiences they have lived through. In their telling of events, matters arise such as helplessness, poverty and suffering throughout the long journey to get to Spain. Many specify that in these stories experiences suffered are detailed, such as: desperation, illnesses during the journey, mistreatment, rape, forced marriage, paedophilia and child abuse, slavery, dispossession and loss of socio-economic status as a result of war, human trafficking, threats due to sexual orientation or ideology, forced separation from loved ones, blackmailing by human traffickers, torture, organ trafficking, summary executions, persecution, racism, prostitution in order to feed their children, etc. 

Regarding the psychological and emotional effects on the interpreters provoked in the episodes mentioned, an extensive list is given, which is broken down below:

It is worth clarifying that in this free response field all of the interpreters referred to various emotions, set out in the above graphic. Although we are aware of the existing similarity between some of these emotions, we preferred to reflect them as they have been indicated by the participants. 

The referred duration of the impact of these emotions is as follows, Table 5:

It is observed that four interpreters indicate that these emotions always accompany them or appear periodically, suggesting the possibility that they have become chronic sequelae (see Table 5). 

### 4.4. Psychological Support for Interpreters in Asylum and Refugee Contexts

A total of 100% state they are not provided with psychological support for interpreting in asylum and refugee contexts.

### 4.5. Perception of Overcoming Emotional Impact

Linking up with the previous section, the participants were asked whether they had the ability to avoid the episodes leading to the emotional impact having an effect on them. Although the answer to this question is partially given in Section 4.4, with 100% of the interpreters admitting to having experienced impactful episodes in the carrying out of their work, this time the aim was to also ascertain the perception they have of their capacity to face such an impact and determine the possible coping strategies they employ. A split response was obtained, with 52.2% (12) who thought they did and 47.8% (11) who did not. In this regard, those who replied in the affirmative (nine women with an average age of 28.1 and three men with an average age of 40.3) were asked to explain their strategies. The literal responses are listed below in order to provide a better understanding of each point of view:

- “I try to think that in life we all have painful stories depending on our contexts, that at the end of the day interpreting is my profession and I should do it to my best ability full stop. I mustn’t allow it to affect me”.
- “I mustn’t take that person’s side, and only concentrate on interpreting what he says”.
- “The strategies I employ go from trying to live a healthy life, in all aspects, talk to my loved ones about my unease (without going into detail about what happened in order to respect the privacy of the users), trying to put to what point my contribution reaches into perspective and not forgetting that at least through my efforts their right to express themselves and understand what’s being communicated to them is guaranteed, learning to disconnect and be aware of the line separating empathy from excessive involvement”.
- “It depends a lot on the subject being dealt with, but for example I try not to look the client in the eyes, I can sometimes get overwhelmed with the language of the eyes, so I choose not to use it to stay more neutral”.
- “Meditation and Yoga”.
- “I try and focus on the interpreting, that is, in the discourse, the vocabulary, transmitting the message correctly, etc.”.
- “Relaxation exercises, looking for pleasant distractions (music), calming the mind focusing on the message”.
- “Separating work from personal life”.
- “Putting the needs of the person who we’re interpreting first. To alleviate the subsequent feeling of sorrow, it helps to speak about what happened with someone with whom this is possible (to respect confidentiality) or in very general terms (and always protecting the privacy of the affected person) with someone you trust”.
- “There are worse things in life. I can’t take people’s problems home with me, I’ve got to limit myself to interpreting the best way possible. If I drift off course psychologically I won’t be able to keep helping these people”.
- “I try to think that my best contribution to them is to interpret in the best way possible, wish them luck and keep progressing in helping more people. Because of the way I am, I’m a very empathetic person, but I was able to achieve the balance of saying I love my job, I want to help these people and to do so I must try and control my emotions. Sometimes in the middle of an interpreting job, I repeat to myself in my mind: this is your job, don’t let it affect you, don’t let it affect you”.
- “Swallow and keep going, as easy and complicated as that. Hold back those emotions however you can, finish the job and then try not to think about it or clear your mind with another task”.

## 5. Discussion

The main results are analysed below. A similar structure to that used previously in the “results” section will be followed.

### 5.1. Frequency of Interpreting and Interpreting Techniques Used in Asylum and Refugee Contexts

The results obtained (see Table 3) tell us that it is a group in which 52.2% work quite frequently in asylum and refugee contexts, which could suppose an aggravating circumstance in cases of the reiterated impact of negative emotions. In this regard, Piqueras Rodríguez et al. [26] (p. 86) hold that negative emotions can become pathological in some individuals due to an imbalance in frequency, intensity, context adaptation, etc.

In addition to the frequency with which it is interpreted in these contexts, the most commonly used interpreting technique (see Table 4) could have a greater emotional impact, since liaison interpreting requires the interpreter to be in situ, located at an intermediate distance from the speakers, and to transfer the message to the two languages, employing note taking in the case of long segments of information, or when there is a wish for short–medium-term memory support. To this we can add the fact that the interpreter in this mode of dialogue normally works alone and that there is usually a constant exchange of information between the two parties. This activity therefore involves a demanding cognitive load, which, combined with other stress factors, may overburden the cognitive efforts of the interpreter.

Furthermore, in the Spanish/Arabic combination, liaison interpreting implies the frequent use of dialectal variants by the parties, requiring the interpreter to understand and speak the dialect in question. When this is not the case, the overload will be greater, reaching the point where interpreting is unachievable when there is no command over the dialectal variant in use. In this sense, it is worth putting the socio-linguistic reality of the Arab world into context, where there is a predominance of diglossia (modern standard Arabic/dialectal variation) or triglossia (cultured modern Arabic/dialectal variations/foreign language) in all areas of life [17] (pp. 348–351). To offer an illustrative example, there may be a case of an asylum seeker who speaks in a dialectal variant—there are six large dialectal families with their respective regional subdivisions—mixed in with expressions and terms from a foreign language—depending on the ex-colony or foreign influence this is usually French, English or Spanish—and he or she presents documentation in standard Arabic to be sight translated [25].

### 5.2. Emotional Impact of Interpreting in Asylum and Refugee Contexts

The results obtained in this section, where 100% of the participants reported having had an emotional impact from interpreting in asylum and refugee contexts, support our initial premise and offer a significant piece of data—perceived, but no less important because of this—regarding the impact of interpreting in these contexts.

The extreme harshness of asylum seekers’ stories is evident, along with their emotional impact on the interpreters who have to translate all of the details, because here details matter and can be determining factors in the granting or refusal of asylum status requested by those they assist [20,21,22,23], amongst others. The results obtained in this section are similar to those reported by Valero-Garcés [15] (p. 149), quoting the study carried out by Baistow [16], expressing that “beneficiaries of the services of interpreters were mainly asylum seekers, refugees, the disabled and immigrants; and for a high percentage these beneficiaries had lived through difficult circumstances such as, for example, separation from the family, physical abuse, war, domestic violence, torture and persecution”.

**Figure 1:**
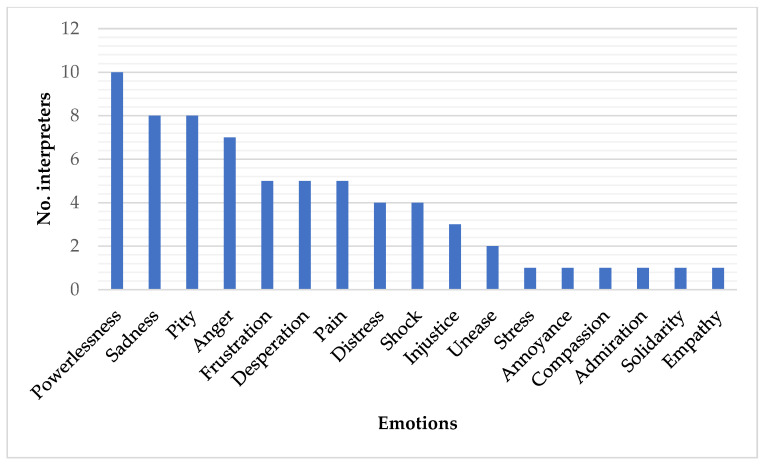
Emotions provoked by the impactful episodes.

Regarding the type of emotions referred to, save for one case of a feeling of admiration and another of solidarity, it is clear that the majority are negative. The most repeated emotion, as can be seen in Figure 1 above, is powerlessness (10 participants), followed by sadness (8), pity (8) and anger (7). There has already been mention in the introductory paragraphs of the psychological effect that these emotions, also referred to as toxic, can cause. This effect may go unnoticed by the interpreter; however, it could interfere in his or her task, lessening cognitive capacity, the quality of work, objectivity, and the complete correct transfer of the message, amongst other parameters. Various authors have pointed to the effect of these negative emotions on the way of acting, patterns of thought and behaviour, and on physical and mental health [26,33,34]. For a list of the physical, psychological and behavioural symptoms that this impact can lead to in interpreters, see Valero-Garcés [15] and Márquez Olalla [14], see Figure 1.

The results obtained contrast in some instances with Baistow [16], where this author finds a generally positive feeling on the part of public service interpreters for the useful work they do. They do compare in terms of the negative effects highlighted by this author, where 49% experienced changes in mood or behaviour. It should not be forgotten that our study is restricted to the context of asylum and refuge, whereas Baistow [16] covered the entire sphere of public services (health, justice, education, etc.).

Another important piece of information for this study was the duration of the impact of these emotions (see Table 5). In this sense, we observe that nearly all participants state it can be anything from the next day, to days, weeks or years afterwards.

The prolonged duration of these emotions is associated with possible pathological behaviours. In this regard, the American Psychiatric Association [29] offers a clinical description of depression as the sensation of sadness, despair, emptiness and loss of interest and pleasure in daily activities for more than two weeks. Piqueras Rodríguez et al. [26] (p. 86), for their part, argue that when the negative impact of emotions “is maintained over a certain time, it can result in a health disorder, both mental (anxiety, major depression, pathological anger, etc.) and physical (cardiovascular, rheumatological, immunological disorders, etc.)”. To obtain an exhaustive scientific description of these negative emotions and their relationship-effect on mental and physical health, see Piqueras Rodríguez et al. [26] (pp. 90 et seq.).

### 5.3. Psychological Support for Interpreters and Perception of Overcoming Emotional Impact in Asylum and Refugee Contexts

The lack of psychological support is noteworthy bearing in mind the consequences of the emotional impact alluded to above. In any event, it all points to the fact that this support is necessary, especially so in the cases of those who experience a prolonged emotional impact over time. In addition, it would be convenient to empower interpreters in this context with strategies and tools to face or mitigate the emotional impact derived from their work. If they cannot deal with such negative emotions, it is possible that their work and mental and physical health will be affected. In this regard, the scarcity or absence of training in stress management is not exclusive to Spanish/Arabic interpreting in contexts of asylum and refuge. In her study on the matter 20 years ago now, Baistow [16] concluded that only 10% of the 295 participating interpreters (France, Netherlands, Germany, Italy, Spain and the United Kingdom) had received training in this area. However, when asked to evaluate emotional and psychological assistance, practically all of them (95%) considered it to be very or quite useful. Regarding the type of support, the majority consider three strategies to be useful: speaking with colleagues, speaking with employers and having access to support groups [15,16] (p. 150).

The initial aim of this study is not the consideration of possible solutions to the emotional impact, but rather to ascertain its possible existence and how and where it manifests itself. However, it may be appropriate in future work to explore possible therapeutic solutions and their effectiveness in the treatment of this impact, especially when the results indicate that psychological support for interpreters in asylum and refuge contexts is non-existent. Taking advantage of the great advances made in neuroendocrinology and psychoneuroimmunology in the 21st century [31], there could be an exploration of cognitive therapy proposals based on mindfulness [37], or cognitive–behavioural group psychotherapy [38], amongst others.

Although it appears that some find channels of distraction (see Section 4.5), such as relaxation and speaking about it to offset the emotional impact, it is noteworthy that several of them admit that this impact is an imposed reality and suppress its possible effects. It would be necessary to put this information into perspective with the other half who do not think they have strategies for coping with the emotional impact generated in this context, which would be a strong reason for offering a solution to this group. To echo Baistow [15,16] (p. 150), the most appropriate strategies according to interpreters are (in this order): speaking about work problems, keeping or increasing social relationships and doing sport and physical exercise.

## 6. Conclusions

In light of the results obtained and their subsequent analysis, it can be concluded that Spanish/Arabic interpreting in asylum and refugee contexts does indeed expose interpreters to a high perceived emotional impact, mainly caused by the harshness of the accounts they interpret. The negative effects referred to are mainly of a cognitive, physiological and behavioural nature, which suggests they may affect not just the interpreting task, but also the physical and mental health of interpreters working in this context, more so when the emotional impact is prolonged over time. In these working settings, it is furthermore concluded that external psychological support is non-existent and it is confirmed that there is a need for specific coping strategies to mitigate the emotional impact caused.

Regarding the participant sample in the study, it is concluded that it is a young group, with a high level of academic training in Translation and Interpreting. Moreover, it is concluded that the group of participants is representative of the group of Spanish/Arabic interpreters in asylum and refugee contexts in Spain, despite not being as numerous as in the mentioned previous literature. This reinforces the validity of the data obtained and encourages the continued exploration of other aspects that arise from this study that it has not been possible to analyse in depth here, as they require further analysis and do not initially form part of the objectives of this paper. Such aspects include the need for therapeutic solutions to mitigate the impact in this context, or the degree to which this emotional impact could affect interpreting.

## Figures and Tables

**Table 1:** Participants’ academic education.

Academic Level	Area of Speciality			
	Translation and Interpreting	Arabic Philology	Hispanic Philology	Others
Degree	4	-	-	Law: 1 Social work: 1
Professional Master’s Degree (+Degree.)	12	1	1	
Ph.D. (+Degree and Master’s)	4	-	-	

**Table 2:** Participants’ language profile.

Language/Interpreters	Arabic	Spanish	French	English	Others
A	9 interpreters	14	-	-	Berber: 1
B	12 (dialectal in 1 case)	19	1	2	-
C	-	1	9	8	Portuguese: 1 German: 1

**Table 3:** Frequency of interpreting in asylum and refugee contexts.

Frequency	Daily	A Number of Times a Week	A Number of Times a Month	A Number of Times a Year	Occasionally (e.g., During Immigration Emergencies)
No. interpreters	9	3	4	3	4

**Table 4:** Interpreting techniques used.

Interpreting Technique Used	No. Interpreters
Liaison in situ	17
Liaison telephone	7
Consecutive (one-directional and with note taking)	2
Sight translation	2
Whispered (chuchotage)	1

**Table 5:** Duration of emotional impact in interpreters.

Duration	No. Interpreters
No further than the act I interpreted	2
Almost the entire following day	3
Days afterwards	5
Weeks	4
Months	1
Years	2
Other…	
At the beginning more than now It keeps repeating	4 4

## Data Availability

Complete data supporting reported results can be found in Spanish (study’s original language) at Figshare dataset https://doi.org/10.6084/m9.figshare.13313231.v2 (accessed on 18 October 2021) [39].
